# Contrasting socioeconomic inequality with noncommunicable diseases: Insights from a population‐based survey using the concentration index in Kong cohort study

**DOI:** 10.1002/hsr2.1682

**Published:** 2023-11-05

**Authors:** Ali Mouseli, Mehdi Sharafi, Zahra Mastaneh, Maryam Shiravani Shiri

**Affiliations:** ^1^ Social Determinants in Health Promotion Research Center, Hormozgan Health Institute Hormozgan University of Medical Sciences Bandar Abbas Iran; ^2^ Department of Health Services Management, School of Health Hormozgan University of Medical Sciences Bandar Abbas Iran; ^3^ Department of Health Information Management and Technology, School of Allied Medical Sciences Hormozgan University of Medical Sciences Bandar Abbas Iran

**Keywords:** Bandar Kong cohort, concentration index, low‐income countries, noncommunicable disease, socioeconomic inequality

## Abstract

**Background:**

Noncommunicable diseases (NCDs) are the major causes of mortality across the globe, which impose a substantial burden on health care systems, particularly in low‐ and middle‐income countries. The present study aimed to determine socioeconomic inequality in the prevalence of NCDs using the concentration index (CI).

**Methods:**

This cross‐sectional study was conducted on the baseline data of the Bandar Kong cohort. The principal component analysis was used to determine people's socioeconomic status (SES). The CI and Lorenz Curve were used for the assessment of socioeconomic inequality. Multivariate logistic regression was used to assess the relationship between SES and the prevalence of NCDs. A *p* Value less than 0.05 is considered significant.

**Results:**

Frequency and prevalence of diabetes was 653 (16.22%), hypertension 848 (21.06%), chronic lung diseases 161 (4%), epilepsy 70 (1.74%), mental disorders 191 (4.74%), stillbirth 299 (13.94%), thyroid disorders 391 (9.71%) and depression 146 (3.63%). CI for the prevalence of diabetes was [−0.107, %95 CI: −0.146 to −0.068], hypertension [−0.122, %95 CI: −0.155 to −0.088], chronic lung disease [−0.116, %95 CI: −0.202 to −0.03], psychiatric disorders [−0.230, %95 CI: −0.304 to −0.155], depression [−0.132, %95 CI: −0.220 to−0.043] and stillbirth [−0.162, %95 CI: −0.220 to −0.105]. The Gini index was negative for all these diseases, indicating that these are significantly concentrated in people of poor SES.

**Conclusions:**

The findings suggest that selected NCDs were concentrated among the poor and the low‐income. Particular attention may be necessary to address the problem of NCDs among these groups.

## 1 INTRODUCTION

Globally, noncommunicable diseases (NCDs) are responsible for the majority of premature deaths and disabilities imposing a substantial burden on the health care advancement of nations. In 2019, NCDs were accountable for 74.4% of all deaths and 63.8% of disability‐adjusted life years (DALYs) on a global scale.^1^ The majority of premature deaths caused by NCDs occur in low‐ and middle‐income countries, including Iran. As a middle‐income country, Iran is particularly vulnerable to the impact of NCDs, which significantly burdens the health care system.^1^, ^2^, ^3^

According to WHO reports in 2020, NCDs were responsible for 82% of deaths in Iran, with cardiovascular diseases (CVD) and cancers being the leading causes, and these diseases accounted for 78.1% of the overall burden of diseases in the country.^4^ NCDs result in reduced health‐related quality of life and increased financial strain.^5^, ^6^

Estimates indicate that the aggregate cost attributed to the five primary NCDs—namely CVD, chronic respiratory disease, cancer, diabetes, and mental health conditions—is projected to amount to an astonishing sum of US$ 47 trillion during the period from 2010 to 2030. This equates to an average annual cost surpassing US$ 2 trillion.^6^

Iran is currently undergoing a significant transitional phase marked by demographic changes that have led to an aging population. This shift, coupled with evolving risk factors for diseases, has resulted in a notable transition from infectious diseases to NCDs. To effectively address this situation, it is crucial to acquire a comprehensive understanding of the risk factors associated with NCDs and formulate practical strategies to minimize their impact.^7^, ^8^

These risk factors can be classified into two categories: modifiable and non‐modifiable. Non‐modifiable factors encompass variables that are beyond an individual's control, such as age, gender, ethnicity, and genetic factors. Conversely, modifiable factors are those that individuals can actively change, such as lifestyle choices, social factors, and cultural influences. Empirical evidence at the global level indicates that NCDs tend to have a greater impact on individuals with lower socioeconomic status (SES) compared to those with higher SES. This disparity is observed both within high‐income nations and across low‐ and middle‐income countries.^9^, ^10^, ^11^

The lower SES population is more prone to experiencing a greater burden of NCDs due to various underlying factors. These factors include material deprivation, psychosocial stress, increased engagement in risky behaviors, adverse living conditions, limited access to high‐quality health care, and diminished opportunities for preventive measures.^12^

Specifically, lower SES groups are more likely to exhibit certain health behaviors that can have negative impacts on their well‐being. These behaviors include a higher tendency to use tobacco products, adopt unhealthy dietary practices, lead sedentary lifestyles, and have a higher prevalence of overweight or obesity.^13^ Disparities in health outcomes have been attributed to inequitable distribution of disease burden or variations in behavioral risk factors which disproportionately impact specific subpopulations.^14^

These inequalities can be quantified across various subgroups of the population, including SES, gender, ethnicity, geographical location, and other relevant factors. Socioeconomic disparity in health is a prevailing perspective in addressing health‐related inequalities. Such inequalities in the socioeconomic aspect of the health care system have a profound impact on overall community health indicators, exacerbating poverty and inequality.^15^ In the context of Iran, limited research has been conducted to assess socioeconomic inequalities in NCDs, particularly studies employing the concentration index (CI) as a measure of inequality. Consequently, this study aimed to examine these inequalities using the CI.

## 2 METHODS

### 2.1 Study population

This cross‐sectional study was conducted on the baseline data of the Bandar Kong cohort. The Bandar Kong cohort is one of the 22 ongoing cohort studies in Iran, which aims to investigate the risk factors of CVD in the age group of 35−70. Bandar Kong is located in the southern part of Iran at 26° 35′ 45.49″ N, 54° 56′ 14.2″ E. This cohort started in October 2016 and its baseline data was collected in 2021. The initial population of this cohort is 4063 people. The cohort profile study of Bandar Kong was previously published.^16^

### 2.2 Measurements

Baseline data of the Bandar Kong cohort is used in this study. This data is collected by interviewers trained by the central team of Iran's cohort.

#### 2.2.1 SES

The SES of people was measured by the asset index. Asset index has fewer fluctuations compared to measurement based on people's income or the amount of people's expenses. Asset index in this study includes variables: owning a house, area of the house, number of rooms, number of household members, having a freezer, dishwasher, washing machine, having a computer, access to the Internet, owning a car, motorcycle, car price, having a bathroom at home, having a vacuum cleaner, having a mobile phone, using a mobile phone, the number of books read, the number of domestic trips; number of foreign trips; The number of pilgrimage trips and have a color TV. The principal component analysis was used to determine people's SES after classification and weighing the asset index variables. After calculating the SES score, people were divided into five quartiles. q1 and q2 placed as poor group, q3 middle, and q4 and q5 were placed in the rich group.^17^

Other measurements include demographic variables, and questionnaires of NCDs including diabetes, blood pressure, chronic lung diseases, thyroid disorders, epilepsy, stillbirth, depression, and mental disorders.

### 2.3 Statistical analysis

#### 2.3.1 CI

**Figure 1:**
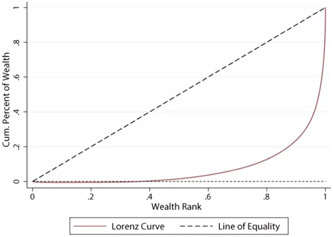
Lorenz curve.

The socioeconomic inequality in NCDs was measured by the CI method, which value is −1 to +1. This index is one of the most common scales of inequality in the field of economy. This index provides the possibility to measure inequality in the health field by respecting the distribution of the health variable in all health classes. The CI is used as a tool to quantify the degree of inequality related to wealth in a health variable and it originates from the concentration curve. A Lorenz curve gives more detailed information about the exact distribution of wealth or income across a health status of a population. Because a Lorenz curve visually displays the distribution across each percentile, it can show precisely at which income (or wealth) percentiles the observed health status varies from the line of equality and by how much. In this curve (Figure 1), the x‐axis (x) is the cumulative percentage of the population under study ranking with the SES, starting from the lowest socioeconomic level and ending at the highest level. And on the y‐axis (y) The cumulative percentage of the health variable (which in this study is NCDs) is related to socioeconomic classes.^18^, ^19^

If the health status is equally distributed in all dimensions of SES, the concentration curve will be diagonally centered, and the equality line 45 (the value of the CI is zero). Suppose the health status is equally distributed in all dimensions of SES. In that case, the concentration curve will be diagonally centered, and the equality line 45° (the value of the CI is zero). If the health status is concentrated in the poor classes of society, the concentration curve will be above the diagonal line (the CI value is negative). The further the concentration curve is from the equality line, the greater degree of inequality. On the other hand, if the concentration curve is below the diagonal line, the unfavorable health situation is concentrated among the wealthy class of society (the CI value is positive) and the further the concentration curve is from the diameter the degree of inequality is greater.^20^

Also, multivariate logistic regression was used to assess the relationship between SES and the prevalence of NCDs. To report the size of the association the Adjusted Odds Ratio (AOR) and %95 Confidence Interval (CI) were used. The frequency and prevalence of diseases were reported based on the quantiles of SES and their relationship was investigated with the chi‐square test. A p‐value less than 0.05 is considered significant.

## 3 RESULTS

The baseline data of 4206 participants in the study showed that the frequency and prevalence of diabetes was 653 (16.22%), hypertension 848 (21.06%), chronic lung diseases 161 (4%), epilepsy (70 (1.74%), mental disorders 191 (4.74%), stillbirth 299 (13.94%), thyroid disorders 391 (9.71%) and depression 146 (3.63%). Table 1 shows the prevalence and frequency of diseases in the overall population and socioeconomic subgroups. The results showed a significant relationship between SES and the prevalence of diabetes, high blood pressure, mental disorders, stillbirth, and depression, *p* < 0.05.

**Table 1:** Frequency and prevalence of noncommunicable diseases by socioeconomic status in Bandar Kong cohort study.

Variable	Subgroup	Poorest quintile	2nd quintile	Middle quintile	4th quintile	Richest quintile	Total	*p* Value
		*N* (%)	*N* (%)	*N* (%)	*N* (%)	*N* (%)	*N* (%)	
Diabetes	No	646 (80.15)	659 (81.86)	665 (82.61)	681 (84.60)	722 (89.69)	3373 (83.78)	<0.001
	Yes	160 (19.85)	146 (18.14)	140 (17.39)	124 (15.40)	83 (10.31)	653 (16.22)	
Has a hypertension	No	581 (72.08)	623 (77.39)	620 (77.02)	660 (81.99)	695 (86.21)	3178 (78.94)	<0.001
	Yes	225 (27.92)	182 (22.61)	185 (22.98)	145 (18.01)	111 (13.79)	848 (21.06)	
Has a chronic lung disease	No	764 (94.79)	768 (95.40)	775 (96.27)	775 (96.27)	783 (97.27)	3865 (96.00)	0.112
	Yes	42 (5.21)	37 (4.60)	30 (3.73)	30 (3.73)	22 (2.73)	161 (4.00)	
Has an epilepsy	No	786 (97.52)	790 (98.14)	796 (98.88)	791 (98.26)	793 (98.51)	3956 (98.26)	0.311
	Yes	20 (2.48)	15 (1.86)	9 (1.12)	14 (1.74)	12 (1.49)	70 (1.74)	
Has psychiatric disorder	no	743 (92.18)	755 (93.79)	771 (95.78)	778 (96.65)	788 (97.89)	3835 (95.26)	<0.001
	yes	63 (7.82)	50 (6.21)	34 (4.22)	27 (3.35)	17 (2.11)	191 (4.74)	
Has stillbirth	No	460 (80.84)	419 (85.34)	366 (85.31)	367 (90.84)	234 (92.86)	1846 (86.06)	<0.001
	Yes	109 (19.16)	72 (14.66)	63 (14.69)	37 (9.16)	18 (7.14)	299 (13.94)	
Has thyroid disease	No	736 (91.32)	714 (88.70)	720 (89.44)	723 (89.81)	742 (92.17)	3635 (90.29)	0.115
	Yes	70 (8.68)	91 (11.30)	85 (10.56)	82 (10.19)	63 (7.83)	391 (9.71)	
Has depression	No	765 (94.91)	772 (95.90)	774 (96.15)	781 (97.02)	788 (97.89)	3880 (96.37)	0.019
	Yes	41 (5.09)	33 (4.10)	31 (3.85)	24 (2.98)	17 (2.11)	146 (3.63)	

The results of the CI (Gini index) by gender and overall population are shown in Table 2. The CI curve (Lorenz Curve) is shown in Figure 2. Based on the results, the CI for the prevalence of diabetes was −0.107 (CI: −0.146 to −0.068) and for men and women, it was −0.050 and −0.109, which shows that this disease is concentrated in the poor group. Figure 2A clearly shows that the inequality line is above 45° line and has a significant difference from the equality line. For the prevalence of hypertension, the CI score was −0.122, which indicates the concentration of the disease in the poor group (Figure 2B).

**Table 2:** Concentration index (Gini index) in noncommunicable diseases in Bandar Kong cohort study.

Variable	Sex	Concentration index	Lowe band 95% CI	Upper band 95% CI
Diabetes	Male	−0.050	−0.116	0.015
	Female	−0.109	−0.156	−0.061
	Population	−0.107	−0.146	−0.068
Has a hypertension	Male	−0.015	−0.075	0.044
	Female	−0.141	−0.181	−0.102
	Population	−0.122	−0.155	−0.088
Has a chronic lung disease	Male	−0.176	−0.327	−0.025
	Female	−0.049	−0.157	0.057
	Population	−0.116	−0.202	−0.03
Has Epilepsy	Male	−0.163	−0.369	0.041
	Female	−0.049	−0.247	0.147
	Population	−0.087	−0.228	0.052
Has psychiatric disorder	Male	−0.180	−0.357	−0.003
	Female	−0.168	−0.250	−0.085
	Population	−0.230	−0.304	−0.155
Has thyroid disease	Male	0.0136	−0.132	0.159
	Female	0.066	0.0120	0.120
	Population	−0.032	−0.084	0.018
Has depression	Male	0.082	−0.150	0.314
	Female	−0.084	−0.179	0.010
	Population	−0.132	−0.220	−0.043
Has stillbirth	−0.162		−0.220	−0.105

Figure 2(A−H) are Lorenz concentrations index that illustrates the socioeconomic inequity in noncommunicable diseases. the 45° line is equality and the red line shows inequality. The greater distance from the equality line, the greater the inequality. If the red line is above the 45° line, it indicates the disease is concentrated in the poor group, and if it is below the 45° line, it shows the concentration of the disease in the rich group. And if it corresponds to the 45° line, it indicates an equal distribution.

**Figure:**
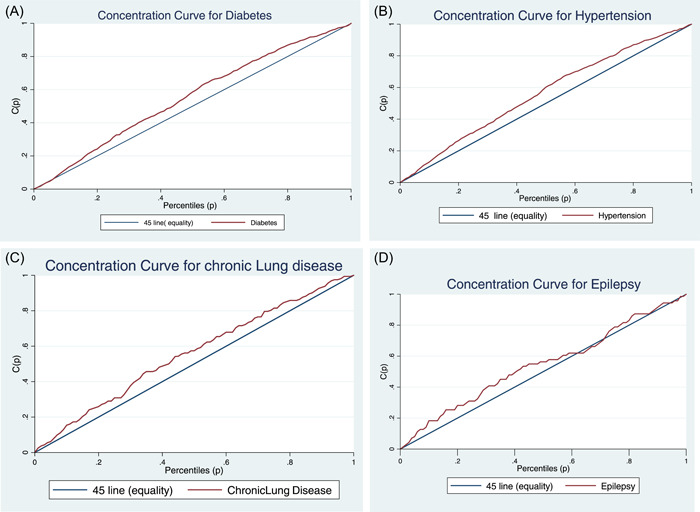

**Figure:**
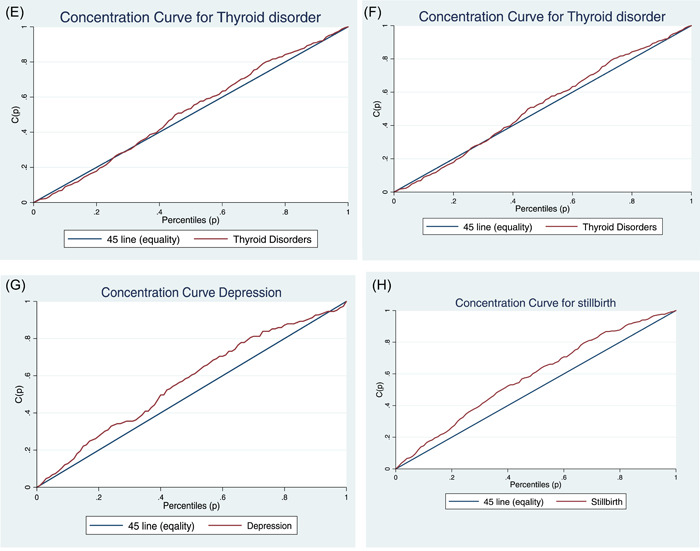

The CI for the prevalence of chronic lung diseases; epilepsy, mental disorders, thyroid disorders, depression, and stillbirth are −0.116, −0.049, −0.230, −0.032, −0.132, and −0.162 respectively. For all these diseases, the concentration is in the poor group. Figure 2C−H, show the concentration of the disease in the poor group.

The result of the multivariate logistic regression model shows that by increasing the socioeconomic level from poor to rich, the odds of having diabetes significantly decreased by 0.33 [AOR: 0.67, %95 CI: 0.55−0.82]. Also, for hypertension, the odds of having hypertension significantly decrease when the socioeconomic level changes from poor to rich [AOR: 0.60, %95 CI: 0.50−0.7]. But for the middle group, no significant relationship was seen. By increasing the SES, the odds of having a psychiatric disorder decrease by 0.36 [AOR: 0.64, 95% CI: 0.43−0.95] and 0.56 [AOR: 0.44, %95 CI: 0.30−0.64] respectively. For Stillbirth, when SES changed from poor to rich, the odds ratio significantly decreased by 0.56 [AOR: 0.44, %95 CI: 0.032−0.61]. No significant relationship was observed for other diseases under study. Table 3 shows the summary results of multivariate logistic regression. The summary results of multivariate logistic regression is shown in Table 3: A1.2.3.

**Table 3:** A1.2.3 summary results of multivariate logistic regression for association between socioeconomic status and noncommunicable diseases in Bandar Kong cohort.

A.1				
Variable	Group	Diabetes	hypertension	Chronic lung disease
		OR_adjust_(%95 CI)	OR_adjust_(%95 CI)	OR_adjust_(%95 CI)
Socioeconomic status	poor	Reference	Reference	**Reference**
	Middle	0.93 (0.78−1.16)	0.92 (0.75−1.12)	0.79 (0.51−1.22)
	Rich	**0.67 (0.55**−**0.82)**	**0.60 (0.50**−**0.72)**	0.71 (0.049−1.03)

A.2				
**Variable**	**Group**	**Thyroid**	**Epilepsy**	**Psychiatric disorder**
		OR _adjust_ (%95 CI)	OR_adjust_ (%95 CI)	OR_adjust_ (%95 CI)
Socioeconomic status	Poor	Reference	Reference	**Reference**
	Middle	1.28 (0.97−1.71)	0.48 (0.23−1.02)	**0.64 (0.43−0.095)**
	Riches	1.29 (1.01−1.64)	0.68 (0.40−1.16)	**0.44 (0.30**−0**.64)**

A.3			
**Variable**	**Group**	**Depression**	**Stillbirth**
		OR_adjust_ (%95 CI)	OR_adjust_ (%95 CI)
Socioeconomic status	poor	Reference	**Reference**
	Middle	0.69 (0.62–1.47)	0.83 (0.61–1.14)
	Riches	0.71 (0.48–1.06)	**0.44 (0.032**–**0.61)**

Abbreviations: OR_adjust_, adjusted odds ratio, %95 CI, %95 confidence interval.

## 4 DISCUSSION

The primary objective of this research was to investigate the disparities associated with socioeconomic factors concerning NCDs. By utilizing a CI analysis, the study determined that individuals of lower SES exhibited higher rates of health conditions, thus indicating the existence of pro‐poor inequalities.

In terms of the CI in the context of NCDs, the highest levels of inequality were observed in psychiatric disorders, followed by stillbirth and depression. These disparities were particularly prominent among individuals who faced economic disadvantages and belonged to lower SES.

In a study conducted by Jafari et al. it was found that adolescents from lower SES backgrounds had a higher prevalence of mental disorders and emotional problems. An analysis of the gap between the two economic groups revealed that factors such as physical activity, school performance, exercise, parents' smoking habits, and gender were identified as the most significant determinants contributing to the observed inequalities.^21^

In a separate investigation conducted on 22,300 households in Tehran in 2007, it was discovered that mental disorders exhibited a negative CI. This implies that individuals with lower SES are disproportionately affected by mental disorders. Additionally, educational attainment (13.4%), age group (13.1%), residential district (12.5%), and employment status (6.5%) emerged as significant contributing factors to the observed inequality.^22^ The presence of a negative CI implies that stillbirths are disproportionately concentrated among individuals with lower SES. This observation has been supported by several other studies conducted in both developed and developing nations.^23^, ^24^, ^25^ However, it is important to note that one study presented conflicting results that contradicted our findings.^26^

Upon conducting a decomposition analysis of the CI, it was revealed that certain factors made substantial positive contributions to the measured inequality in the occurrence of stillbirths in Tehran. Notably, mother's education accounted for 50% of the observed inequality, followed by mother's occupation (30%), economic status (26%), and father's age (12%). Conversely, the mother's age exhibited the highest negative contribution to inequality, accounting for 17% of the measured disparity.^23^ Furthermore, a global study indicated that a significant majority of stillbirths, approximately 98%, occur in low‐income and middle‐income countries, with 77% of cases concentrated in South Asia and sub‐Saharan Africa.^24^

Our findings are in line with previous evidence indicating a negative correlation between hypertension and SES. This relationship has been commonly observed in developed countries and nations with higher per capita incomes.^27^, ^28^ However, it is important to note that the study conducted by Lai et al. yielded contradictory results, deviating from our observations.^29^ Research has demonstrated that individuals from economically disadvantaged backgrounds exhibit lower dietary intakes aimed at preventing chronic diseases such as high blood pressure. This can be attributed to several factors. Firstly, individuals with limited financial resources are less inclined to purchase nutritious foods.^30^

Additionally, they are disproportionately located in areas with restricted access to fresh and healthy food options. Furthermore, individuals from economically disadvantaged backgrounds are less likely to purchase foods with low salt content or engage in salt restriction practices.^31^, ^32^ Furthermore, in accordance with our findings, the prevalence of lung diseases was found to be disproportionately higher among individuals with low SES, thus indicating the presence of pro‐poor inequality. This observation aligns with the results of prior investigations conducted in similar contexts.^17^, ^33^

Moreover, our study identified a pro‐poor socioeconomic inequality specifically concerning diabetes in Iran, which is consistent with the findings of earlier studies conducted in comparable settings.^34^, ^35^ Individuals of lower SES may face challenges in affording adequate nutrition and health care services, consequently impacting their access to such resources as well as their health‐seeking behaviors, including costs related to transportation, travel, and potential work absences.^33^, ^36^ Interestingly, certain studies have documented a greater concentration of diabetes burden in countries with higher SES, presenting a contrasting trend.^20^, ^37^, ^38^ Within the context of this study, the CI was not statistically significant for epilepsy and thyroid disease; however, the negative value of the CI indicates a higher prevalence of these diseases among households with lower SES.

According to multivariate logistic regression, there was a significant reduction in the prevalence of diabetes, hypertension, psychiatric disorders, and stillbirths among the wealthy groups. In other words, with improving socioeconomic indicators, the prevalence of these diseases reduced significantly. A notable strength of this study was the utilization of robust Kong cohort data, obtained through rigorous and standardized methodologies, offering a substantial sample size. Nonetheless, the cross‐sectional design employed in this study posed a limitation. In such observational studies, the interpretation of associations between variables should be approached with caution, as they may not necessarily imply causality.

## 5 CONCLUSION

In our analysis, we observed a statistically significant and negative CI for psychiatric disorders, stillbirth, depression, hypertension, lung diseases, and diabetes. This indicates a higher concentration of these diseases among individuals with low SES and highlights the presence of pro‐poor inequality. However, the CI values for epilepsy and thyroid disorder were not statistically meaningful. Identifying the factors that influence the occurrence of NCDs within communities is crucial for developing effective prevention and control policies. Consequently, assessing the socioeconomic determinants of NCDs holds significant importance.

## AUTHOR CONTRIBUTIONS

**Ali Mouseli**: Conceptualization; resources; supervision; writing—original draft. **Mehdi Sharafi**: Conceptualization; methodology; supervision; writing—original draft. **Zahra Mastaneh**: Validation; writing—original draft. **Maryam Shiravani Shiri**: Writing—original draft.

## CONFLICT OF INTEREST STATEMENT

The authors declare no conflict of interest.

## ETHICS STATEMENT

PERSIAN Cohort Study was approved by the ethics committees of the Ministry of Health and Medical Education Fasa is one of the regions. This study is in agreement with the Helsinki Declaration for observational studies and Iranian national guidelines for ethics in research. and informed written consent was obtained from all participants. This study was also approved by the Research Ethics Committee of Hormozgan University of Medical Sciences (ID: IR.HUMS.REC.1402.282).

## TRANSPARENCY STATEMENT

The lead author Mehdi Sharafi, Zahra Mastaneh affirms that this manuscript is an honest, accurate, and transparent account of the study being reported; that no important aspects of the study have been omitted; and that any discrepancies from the study as planned (and, if relevant, registered) have been explained.

## Data Availability

The datasets generated and/or analyzed during the current study are not publicly available due to their being the intellectual property of Hormozgan University of Medical Sciences but are available from the corresponding author upon reasonable request.
